# Chemotherapy alone versus definitive concurrent chemoradiotherapy for cT4b esophageal squamous cell carcinoma: a population-based study

**DOI:** 10.1186/s12876-021-01742-4

**Published:** 2021-04-07

**Authors:** Chia-Chin Li, Chih-Yi Chen, Ying-Hsiang Chou, Chih-Jen Huang, Hsiu-Ying Ku, Chun-Ru Chien

**Affiliations:** 1 Department of Radiation Oncology, China Medical University Hsinchu Hospital, Hsinchu, Taiwan; 2 Division of Thoracic Surgery, Department of Surgery, Chung Shan Medical University Hospital, Taichung, Taiwan; 3 Institute of Medicine, Chung Shan Medical University, Taichung, Taiwan; 4 Department of Medical Imaging and Radiological Sciences, Chung Shan Medical University, Taichung, Taiwan; 5 Department of Radiation Oncology, Chung Shan Medical University Hospital, Taichung, Taiwan; 6 Department of Radiation Oncology, Kaohsiung Medical University Hospital, Kaohsiung, Taiwan; 7 National Institute of Cancer Research, National Health Research Institutes, Miaoli, Taiwan; 8 Department of Healthcare Administration, Asia University, Taichung, Taiwan; 9 Department of Radiation Oncology, China Medical University Hospital, Taichung, Taiwan; 10 School of Medicine, College of Medicine, China Medical University, No. 91 Hsueh-Shih Road, North District, Taichung, 40402 Taiwan

**Keywords:** Chemotherapy, Definitive concurrent chemoradiotherapy, Esophageal squamous cell carcinoma

## Abstract

**Background:**

The role of radiotherapy for cT4bNanyM0 esophageal squamous cell carcinoma (ESqCC) is relatively unclear, with both chemotherapy (C/T) alone and definitive concurrent chemoradiotherapy (dCCRT) being treatment options in the current guidelines. We aimed to compare the survival of dCCRT versus C/T for these patients via a population-based approach.

**Methods:**

Eligible cT4b ESqCC patients diagnosed between 2011 and 2017 were identified via the Taiwan Cancer Registry. We used propensity score (PS) weighting to balance the observable potential confounders between groups. The hazard ratio (HR) of death and incidence of esophageal cancer mortality (IECM) were compared between dCCRT and C/T. We also evaluated OS in subgroups of either low or standard radiotherapy doses.

**Results:**

Our primary analysis consisted of 247 patients in whom covariates were well balanced after PS weighing. The HR for death when dCCRT was compared with C/T was 0.36 (95% confidence interval 0.24–0.53, *P* < 0.001). Similar results were found for IECM. Statistical significance was only observed in the standard RT dose but not in the low dose in subgroup analyses.

**Conclusions:**

In this population-based nonrandomized study of cT4bNanyM0 ESqCC patients from Asia (Taiwan), we found that the use of radiotherapy with chemotherapy was associated with better overall survival than chemotherapy alone. Further studies (especially RCTs) are needed to confirm our findings.

**Supplementary Information:**

The online version contains supplementary material available at 10.1186/s12876-021-01742-4.

## Background

Esophageal cancer is one of the leading cancer deaths worldwide, including in Taiwan [1]. The predominant histology was adenocarcinoma in Western countries and squamous cell carcinoma in Asians [1, 2].

For locally advanced esophageal squamous cell carcinoma (LA-ESqCC), radiotherapy is an important treatment modality [3–5]. However, the role of radiotherapy for cT4bNanyM0 is relatively unclear. Both chemotherapy (C/T) alone and definitive concurrent chemoradiotherapy (dCCRT) are treatment options for cT4b ESqCC in the current North American guidelines [3]. This is possibly related to concerns over radiotherapy-related complications for cT4b disease [6].

Due to the above concerns over the use of radiotherapy for cT4b LA-ESqCC and few relevant studies [7], our study aimed to compare the survival of chemotherapy alone versus definitive concurrent chemoradiotherapy for cT4bNanyM0 esophageal squamous cell carcinoma patients via a population-based approach.

## Methods

### Data

We used the Taiwan Cancer Registry (TCR) as the data source in this study. The quality of TCR was reported to be one of the highest-quality cancer registries in the world [8, 9].

### Study population

We identified esophageal cancers diagnosed between 2011 and 2017 from TCR. The inclusion criteria of our study were (a) ESqCC patients with clinical stage cT4bNanyM0 by the 7th American Joint Committee on Cancer (AJCC); (b) age 20–75 years old; and (c) patients treated with either C/T without radiotherapy or surgery (C/T group) or CCRT without surgery (dCCRT group) according to records in TCR. In the dCCRT group, we only included those who received a conventional fractionated external beam radiotherapy dose ≤ 70 Gy [5, 10, 11]. The exclusion criteria were (a) those with multiple treatment records in TCR and (b) those with prior cancer(s). These inclusion/exclusion criteria were modified from a relevant ongoing trial [12].

### Covariates

We included the following covariates as modified from recent relevant studies and our clinical and research experiences [7, 12–14]. Patient demographics (age, sex, residency), patient characteristics [body mass index (BMI), drinking, smoking], disease characteristics (N-stage, tumor location), and the use of positron emission tomography (PET) were defined as follows. Patient residency region was classified as ‘northern Taiwan’ or ‘non-north’. Smoking, drinking and the use of PET were classified as yes or no. The clinical N-stage was classified as ‘0’ or ‘1–3’. Tumor location was classified as ‘cervical’ or ‘noncervical’.

### Statistical and subgroup analyses

The primary outcome of interest was overall survival (OS). We also evaluated the impact of intervention (C/T vs dCCRT) on the incidence of esophageal cancer mortality (IECM). We adopted the propensity score (PS) approach and used PS weighting (PSW) as the framework for analyses, as advocated in the literature [15–17]. We estimated the probability of receiving dCCRT (vs. C/T) with a logistic regression model based on all the above covariates (i.e., age, sex, residency, BMI, drinking, smoking, N-stage, tumor location, and the use of PET) and then assessed the balance of covariates between groups after PSW using overlap weight [18, 19] via the standardized difference (SDif) [15, 20, 21]. We compared the hazard ratio (HR) of death between the dCCRT group and C/T group groups during the entire follow-up period via the Cox proportional hazards model in the weighted sample for point estimation and used the bootstrap method to estimate the 95% confidence interval (95% CI) [18, 22, 23]. We used the E-value to assess the robustness of our finding regarding potential unmeasured confounder(s), as suggested in the literature [24–26], because the PS approach can only be valid under the assumption of no unmeasured confounder(s). We took a competing risk approach to compare IECM between groups [27]. We performed two separate PSW subgroup analyses (SA) according to the radiotherapy dose (< 50 Gy vs 50–70 Gy) because 50 Gy was the recommended minimal radiotherapy dose for dCCRT in the treatment guidelines [3–5].

## Results

### Study population

**Fig. 1:**
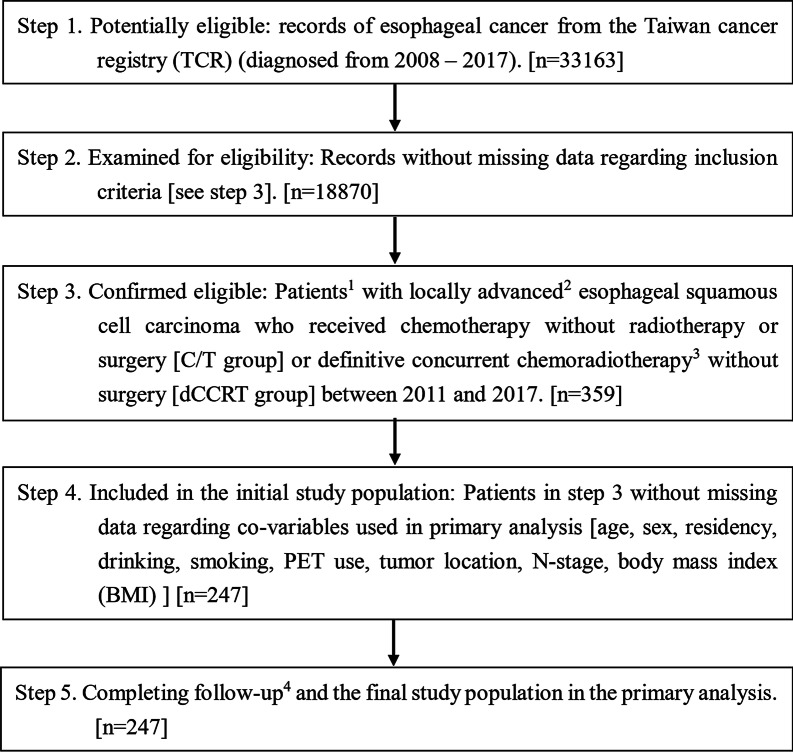
STROBE study flowchart and the number of individuals at each stage of the study. ^1^We only included those treated (classes 1–2) with only one record to ensure data consistency. ^2^The Seventh American Joint Committee on Cancer staging clinical stage cT4bNanyM0. ^3^Conventional fractionated external beam radiotherapy dose ≤ 70 Gy at 1.8–2 Gy/fraction. ^4^Without missing information in the TCR and death registry

We identified 247 patients (56 for the C/T group and 191 for the dCCRT group) as our primary study population, as depicted in Fig. 1 [STROBE]. We achieved covariate balance after PSW, although some imbalance was seen before PSW, as shown in Table 1. After a median follow-up of 7 months (range 0.4–107), death occurred in 56 patients in the C/T group and 173 patients in the dCCRT group. The median follow-up for survivors was 61 months (range 29–107).

**Table 1:** Patient characteristics of the study population in the primary analysis

	C/T Group (n = 56)		dCCRT Group (n = 191)		Standardized difference (rounded)^a^	
	Number or mean (SD)^a^	(%)^a^	Number or mean (SD)^a^	(%)^a^	Before PSW	After PSW
Age (year)	56.11 (9.54)		55.87 (8.16)		0.026	≈ 0
*Sex*						
Female	1	(2)	10	(5)	0.188	≈ 0
Male	55	(98)	181	(95)		
*Residency*						
Non-north	39	(70)	123	(64)	0.112	≈ 0
North	17	(30)	68	(36)		
*Drinking*						
No	14	(25)	20	(10)	0.387	≈ 0
Yes	42	(75)	171	(90)		
*Smoking*						
No	6	(11)	17	(9)	0.061	≈ 0
Yes	50	(89)	174	(91)		
*PET*						
No	41	(73)	67	(35)	0.828	≈ 0
Yes	15	(27)	124	(65)		
*Tumor location*						
Non-cervical	51	(91)	163	(85)	0.178	≈ 0
Cervical	5	(9)	28	(15)		
*N-stage*						
0	4	(7)	17	(9)	0.065	≈ 0
1–3	52	(93)	174	(91)		
BMI	19.30 (3.16)		20.75 (3.69)		0.424	≈ 0

*BMI* body mass index, *C/T* chemotherapy, *dCCRT* definitive concurrent chemoradiotherapy, *PET* positron emission tomography, *PSW* propensity-score weighting, *SD* standard deviation

^a^Rounded

### Primary analysis

**Fig. 2:**
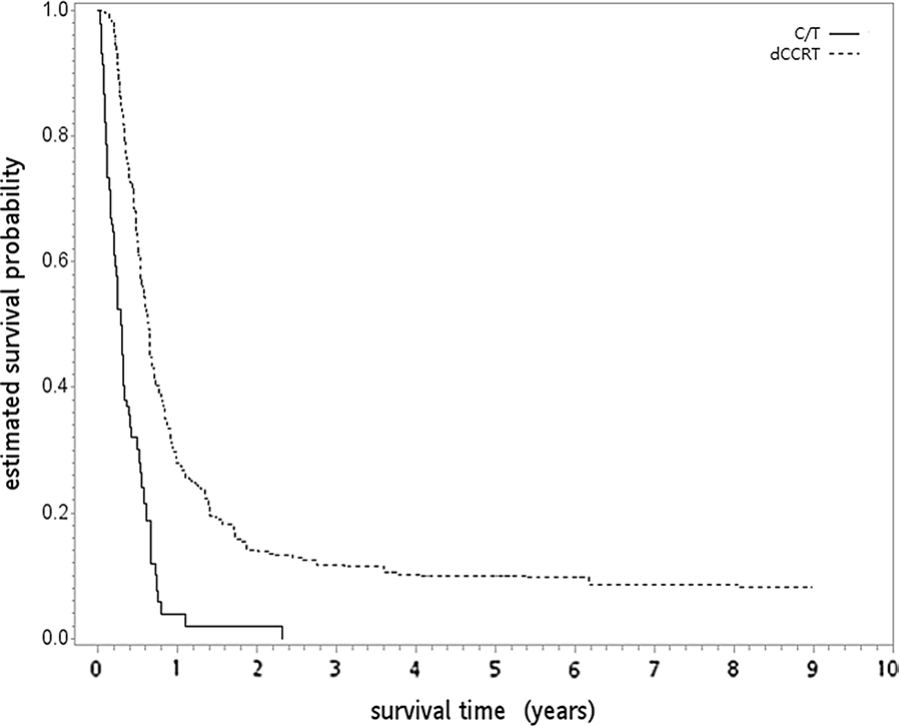
The overlap weight-adjusted overall survival curve (in years) in the primary analysis. *C/T* chemotherapy, *dCCRT* definitive concurrent

The overlap weight-adjusted OS curves are shown in Fig. 2. The 1/2/5-year OS rates for both groups were 4/2/0% (C/T group) and 28/14/10% (dCCRT group), respectively. The median OS (month) was 4 for the C/T group and 8 for the dCCRT group. When the dCCRT group was compared to the C/T group, the HR of death was 0.36 [95% confidence interval (95% CI) 0.24–0.53, *P* ≤ 0.001]. The observed HR of 0.36 for OS could be explained by an unmeasured confounder that was associated with both selections of treatment (C/T vs dCCRT) and outcome (live vs death) by a risk ratio of 3.44 (E-value) fold each, but weaker confounding could not do so [26]. The HR for IECM was 0.49 (95% CI 0.29–0.83, *P* = 0.007).

### Subgroup analyses

In both SA-1 and SA-2, we achieved covariate balance after PSW, although some imbalance was seen before PSW, as shown in Tables 2 and 3. Comparisons between the dCCRT group and the C/T group revealed significantly better OS for dCCRT patients with a standard dose (≥ 50 Gy) but not for those with a low dose (< 50 Gy); the HR of death is summarized in Table 4. For those who received a standard dose (≥ 50 Gy), the rate of death within 3 months of completing RT was 3.7%.

**Table 2:** Patient characteristics in SA-1: dCCRT with a low dose (< 50 Gy)

	C/T (n = 56)		dCCRT with low dose [< 50 Gy] (n = 29)		Standardized difference (rounded)^a^	
	Number or mean (SD)^a^	(%)^a^	Number or mean (SD)^a^	(%)^a^	Before PSW	After PSW
Age (year)	56.11 (9.54)		54.86 (6.90)		0.150	≈ 0
*Sex*						
Female	1	(2)	3	(10)	0.364	≈ 0
Male	55	(98)	26	(90)		
*Residency*						
Non-north	39	(70)	19	(66)	0.088	≈ 0
North	17	(30)	10	(34)		
*Drinking*						
No	14	(25)	2	(7)	0.510	≈ 0
Yes	42	(75)	27	(93)		
*Smoking*						
No	6	(11)	1	(3)	0.286	≈ 0
Yes	50	(89)	28	(97)		
*PET*						
No	41	(73)	8	(28)	1.026	≈ 0
Yes	15	(27)	21	(72)		
*Tumor location*						
Non-cervical	51	(91)	26	(90)	0.048	≈ 0
cervical	5	(9)	3	(10)		
*N-stage*						
0	4	(7)	1	(3)	0.166	≈ 0
1–3	52	(93)	28	(97)		
BMI	19.30 (3.16)		20.83 (3.91)		0.430	≈ 0

*BMI* body mass index, *C/T* chemotherapy, *dCCRT* definitive concurrent chemoradiotherapy, *PET* positron emission tomography, *PSW* propensity-score weighting, *SA* subgroup analyses, *SD* standard deviation

^a^Rounded

**Table 3:** Patient characteristics in SA-2: dCCRT with a standard dose (50–70 Gy)

	C/T (n = 56)		dCCRT with standard dose [50–70 Gy] (n = 162)		Standardized difference (rounded)^a^	
	Number or mean (SD)^a^	(%)^a^	Number or mean (SD)^a^	(%)^a^	Before PSW	After PSW
Age (year)	56.11 (9.54)		56.06 (8.37)		0.006	≈ 0
*Sex*						
Female	1	(2)	7	(4)	0.148	≈ 0
Male	55	(98)	155	(96)		
*Residency*						
Non-north	39	(70)	104	(64)	0.116	≈ 0
North	17	(30)	58	(36)		
*Drinking*						
No	14	(25)	18	(11)	0.367	≈ 0
Yes	42	(75)	144	(89)		
*Smoking*						
No	6	(11)	16	(10)	0.028	≈ 0
Yes	50	(89)	146	(90)		
*PET*						
No	41	(73)	59	(36)	0.796	≈ 0
Yes	15	(27)	103	(64)		
*Tumor location*						
Non-cervical	51	(91)	137	(85)	0.200	≈ 0
cervical	5	(9)	25	(15)		
*N-stage*						
0	4	(7)	16	(10)	0.098	≈ 0
1–3	52	(93)	146	(90)		
BMI	19.30 (3.16)		20.74 (3.66)		0.422	≈ 0

*BMI* body mass index, *C/T* chemotherapy, *dCCRT* definitive concurrent chemoradiotherapy, *PET* positron emission tomography, *PSW* propensity-score weighting, *SA* subgroup analyses, *SD* standard deviation

^a^Rounded

**Table 4:** The HR of death for dCCRT versus C/T

dCCRT versus C/T	Primary analyses: dCCRT (dose ≤ 70 Gy)	SA-1: dCCRT with low dose (< 50 Gy)	SA-2: dCCRT with standard dose (50–70 Gy)
HR	0.36	1.1	0.31
95% CI	0.24–0.53	0.56–2.15	0.21–0.48
*P* value	< 0.001	0.79	< 0.001

*CI* confidence interval, *C/T* chemotherapy, *dCCRT* definitive concurrent chemoradiotherapy, *HR* hazard ratio

## Discussion

In this population-based nonrandomized study of cT4bNanyM0 esophageal squamous cell carcinoma patients from Asia (Taiwan), we found that the use of radiotherapy with chemotherapy was associated with better overall survival than chemotherapy alone. To our knowledge, this is the 1st study on this topic.

A similar trend regarding the role of radiotherapy in these patients was reported in a North American cancer registry-based study in 2019 [7]. The reported median OS for C/T and chemoradiotherapy was 6 and 12.7 months, respectively. However, this study included both SqCC and adenocarcinoma patients, and relevant results specific to SqCC were not reported. We further searched in Dec 2020 using the keywords “((esophageal squamous cell carcinoma) AND (cT4b))” in PubMed but found no additional relevant studies.

The interpretation of our study seems straightforward due to the potential role of radiotherapy in definitive treatment for LA-ESqCC, as observed in previous randomized controlled trials (RCTs) [28, 29]. However, our study somehow relieved the concern for OS (although the concern for toxicity remained) after radiotherapy for this specific population [cT4b], as reflected in the current North American guidelines [3]. However, our study should also be interpreted with caution given its nonrandomized nature, and RCTs are needed for confirmation. However, no RCTs were included in a recent relevant systematic review [30]. When we further searched the trial registry (https://clinicaltrials.gov/) in Dec 2020, we did not find relevant RCTs. Therefore, we believe our study provides useful evidence regarding radiotherapy for cT4bNanyM0 ESqCC while more studies on this topic are awaited.

There were also limitations in our study. First, as with all nonrandomized studies, potential unmeasured confounder(s) such as patient performance status, biomarkers [31] or radiotherapy tolerability were not available due to data limitations, although we used the PS approach to balance observed covariates and reported the E-value to assess the potential impact of potential unmeasured confounder(s). Second, cT4b patients were not a homogenous population. Some subgroups, such as those with vertebral body invasion, may not be the ideal study population [3], but this could not be clarified in our study due to the retrospective nature and data limitations. Third, the use of salvage therapy may have impacted our primary endpoint (OS) but could not be evaluated due to data limitations in the TCR. Fourth, some researchers used neoadjuvant C/T 1st, followed by planned local treatment (usually surgery for those responsive and resectable, or CCRT for the others) [12, 32]. This strategy was not recommended by the North American treatment guidelines for cT4b ESqCC [3] and may not lead to significantly better outcomes (see the Additional file 1 and the Additional file 2), although the results from ongoing RCTs are eagerly awaited [12]. However, due to data limitations, our study was unable to exactly exclude those who were planned for this neoadjuvant C/T strategy but did not take local treatment (probably due to poor response on neoadjuvant C/T), so our results in the C/T group may be biased and underestimated. Fifth, other endpoints [such as quality of life or toxicity (especially fistula) in addition to OS used in our study] might also be relevant, but these were not included in our study due to data limitations. Finally, this study was based on patients treated in Taiwan within the period from 2011 to 2017, so the implications for other population(s) with different covariate distributions are not clear. Furthermore, the impact of new systemic therapies, such as immunotherapy, could not be evaluated [33].

## Conclusions

In this population-based nonrandomized study of cT4bNanyM0 esophageal squamous cell carcinoma patients from Asia (Taiwan), we found that the use of radiotherapy with chemotherapy was associated with better overall survival than chemotherapy alone. Further studies (especially RCTs) are needed to confirm our findings.

## Supplementary Information

**Additional file 1:** Patient characteristics of the study population when nCT was compared to dCCRT.

**Additional file 2:** The overlap weight-adjusted overall survival curve (in years) when nCT was compared to dCCRT for patients in Additional file 1.

## Data Availability

The analyzed datasets of this study are not publicly available due to restrictions apply and will not be made available from the corresponding author on request.

## Abbreviations

AJCC: American Joint Committee on Cancer
BMI: Body mass index
C/T: Chemotherapy
dCCRT: Definitive concurrent chemoradiotherapy
HR: Hazard ratio
IECM: Incidence of esophageal cancer mortality
LA-ESqCC: Locally advanced esophageal squamous cell carcinoma
OS: Overall survival
PET: Positron emission tomography
PS: Propensity score
PSW: Propensity score weighting
SA: Subgroup analyses
SDif: Standardized difference
TCR: Taiwan Cancer Registry
95% CI: 95% Confidence interval
