# Supplementary material for: Chemotherapy alone versus definitive concurrent chemoradiotherapy for cT4b esophageal squamous cell carcinoma: a population-based study
Source: BMC Gastroenterol. 2021 Apr 7;21:153. doi: 10.1186/s12876-021-01742-4 (PMC8028221; doi:10.1186/s12876-021-01742-4)
Supplement: Supplementary file 1 — Additional file 1: Patient characteristics of the study population when nCT was compared to dCCRT. [file 12876_2021_1742_MOESM1_ESM.docx]

Supplementary information file for “Chemotherapy alone vs definitive concurrent chemoradiotherapy for cT4b esophageal squamous cell carcinoma: a population-based study” as suggested during revision

Table S-1. Patient characteristics of the study population when nCT was compared to dCCRT

|  |  | nCT^+^ Group  (n=32) | | dCCRT Group  (n=191) | | Standardized difference  (rounded)^‡^ | |
| --- | --- | --- | --- | --- | --- | --- | --- |
|  |  | Number or mean (SD)^‡^ | (%)^‡^ | Number or mean (SD)^‡^ | (%)^‡^ | Before  PSW | After  PSW |
| Age (year) |  | 57.28 (8.90) |  | 55.87 (8.16) |  | 0.165 | ≈ 0 |
| Sex | Female | 1 | (3) | 10 | (5) | 0.106 | ≈ 0 |
|  | Male | 31 | (97) | 181 | (95) |  |  |
| Residency | Non-north | 23 | (72) | 123 | (64) | 0.161 | ≈ 0 |
|  | North | 9 | (28) | 68 | (36) |  |  |
| Drinking | No | 5 | (16) | 20 | (10) | 0.153 | ≈ 0 |
|  | Yes | 27 | (84) | 171 | (90) |  |  |
| Smoking | No | 2 | (6) | 17 | (9) | 0.100 | ≈ 0 |
|  | Yes | 30 | (94) | 174 | (91) |  |  |
| PET | No | 17 | (53) | 67 | (35) | 0.370 | ≈ 0 |
|  | Yes | 15 | (47) | 124 | (65) |  |  |
| Tumor location | non-cervical | 28 | (87) | 163 | (85) | 0.063 | ≈ 0 |
|  | cervical | 4 | (13) | 28 | (15) |  |  |
| N-stage | 0 | 2 | (6) | 17 | (9) | 0.100 | ≈ 0 |
|  | 1 – 3 | 30 | (94) | 174 | (91) |  |  |
| BMI |  | 20.55 (2.73) |  | 20.75 (3.69) |  | 0.062 | ≈ 0 |

BMI: body mass index; C/T: chemotherapy; CCRT: concurrent chemoradiotherapy; dCCRT: definitive CCRT (without nCT); nCT: neoadjuvant chemotherapy followed by local therapy^+^; PET: positron emission tomography; PSW: propensity-score weighting; SD: standard deviation; ^+^: either esophagectomy or CCRT (but we only identified one patient who received esophagectomy but was not included in table S-1 due to missing information in covariates); ^‡^: rounded.

Figure S-1. The overlap weight-adjusted overall survival curve (in years) when nCT was compared to dCCRT for patients is shown in Table S-1.

95% CI: 95% confidence interval; dCCRT: concurrent chemoradiotherapy (without nCT); HR: hazard ratio of death; nCT: neoadjuvant chemotherapy followed by local therapy.
