# Supplementary figures and images for: Chemotherapy alone versus definitive concurrent chemoradiotherapy for cT4b esophageal squamous cell carcinoma: a population-based study
Source: BMC Gastroenterol. 2021 Apr 7;21:153. doi: 10.1186/s12876-021-01742-4 (PMC8028221; doi:10.1186/s12876-021-01742-4)

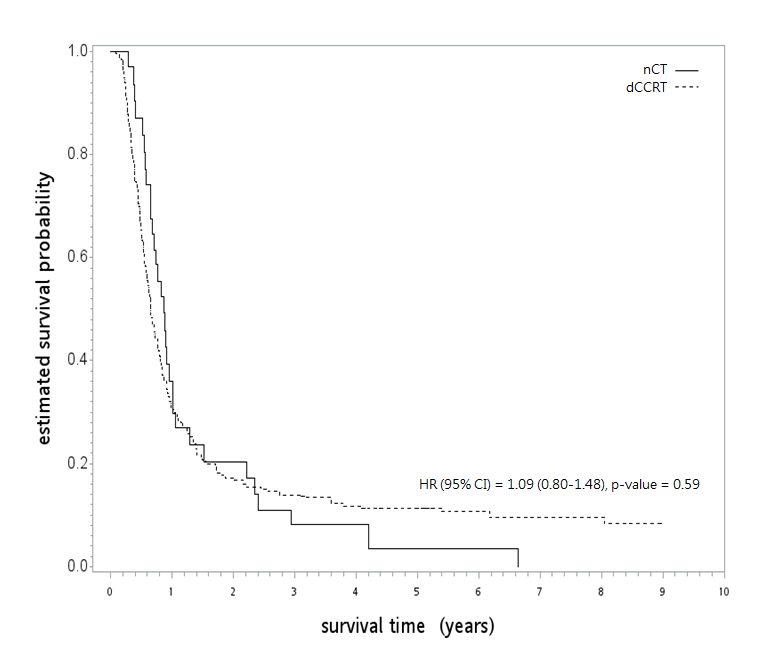

Supplement: Supplementary file 2 — Additional file 2: The overlap weight-adjusted overall survival curve (in years) when nCT was compared to dCCRT for patients in Additional file 1. [file 12876_2021_1742_MOESM2_ESM.tiff]
